# Elevated Protein Carbonylation, and Misfolding in Sciatic Nerve from *db/db* and *Sod1^−/−^* Mice: Plausible Link between Oxidative Stress and Demyelination

**DOI:** 10.1371/journal.pone.0065725

**Published:** 2013-06-04

**Authors:** Ryan T. Hamilton, Arunabh Bhattacharya, Michael E. Walsh, Yun Shi, Rochelle Wei, Yiqiang Zhang, Karl A. Rodriguez, Rochelle Buffenstein, Asish R. Chaudhuri, Holly Van Remmen

**Affiliations:** 1 Department of Cellular and Structural Biology, University of Texas Health Science Center, San Antonio, Texas, United States of America; 2 Department of Physiology, University of Texas Health Science Center, San Antonio, Texas, United States of America; 3 Department of Biochemistry, University of Texas Health Science Center, San Antonio, Texas, United States of America; 4 Sam and Ann Barshop Institute for Longevity and Aging Studies, San Antonio, Texas, United States of America; 5 Geriatric Research Education and Clinical Center, South Texas Veterans Health Care System, San Antonio, Texas, United States of America; Research Inst. of Environmental Med., Nagoya Univ., Japan

## Abstract

Diabetic peripheral polyneuropathy is associated with decrements in motor/sensory neuron myelination, nerve conduction and muscle function; however, the mechanisms of reduced myelination in diabetes are poorly understood. Chronic elevation of oxidative stress may be one of the potential determinants for demyelination as lipids and proteins are important structural constituents of myelin and highly susceptible to oxidation. The goal of the current study was to determine whether there is a link between protein oxidation/misfolding and demyelination. We chose two distinct models to test our hypothesis: 1) the leptin receptor deficient mouse (*dbdb*) model of diabetic polyneuropathy and 2) superoxide dismutase 1 knockout (*Sod1^−/−^*) mouse model of *in vivo* oxidative stress. Both experimental models displayed a significant decrement in nerve conduction, increase in tail distal motor latency as well as reduced myelin thickness and fiber/axon diameter. Further biochemical studies demonstrated that oxidative stress is likely to be a potential key player in the demyelination process as both models exhibited significant elevation in protein carbonylation and alterations in protein conformation. Since peripheral myelin protein 22 (PMP22) is a key component of myelin sheath and has been found mutated and aggregated in several peripheral neuropathies, we predicted that an increase in carbonylation and aggregation of PMP22 may be associated with demyelination in *dbdb* mice. Indeed, PMP22 was found to be carbonylated and aggregated in sciatic nerves of *dbdb* mice. Sequence-driven hydropathy plot analysis and *in vitro* oxidation-induced aggregation of purified PMP22 protein supported the premise for oxidation-dependent aggregation of PMP22 in *dbdb* mice. Collectively, these data strongly suggest for the first time that oxidation-mediated protein misfolding and aggregation of key myelin proteins may be linked to demyelination and reduced nerve conduction in peripheral neuropathies.

## Introduction

Diabetic peripheral polyneuropathy is a consequence of elevated blood glucose leading to pain and dysfunction of lower extremities and potentially loss of limb. In humans, the pathologic changes include neuronal/Schwann cell dysfunction, axonal degeneration and chronic motor/sensory neuron demyelination [1]. Reduced nerve conduction velocity (NCV) is also observed in diabetic mice [2] and in human type II diabetics [3]. Considerable evidence exists to suggest that oxidative stress may play a critical role in reduction of sciatic nerve conduction and alteration of sciatic/myelin morphology and function in diabetes [4], [5], [6]; for example, rodent models of type I and II diabetes show increase in oxidative stress and/or damage, e.g. superoxide production, nitrotyrosine and 4-hydroxy nonenal [7], [8], [9], [10]. However, direct evidence for this hypothesis is still lacking in the literature.

Myelin membrane is produced by Schwann cells and it is rich in cholesterol, lipid and protein [11]. In particular, peripheral myelin membrane has heterogeneous kinds of myelin basic proteins and glycoproteins. Therefore, it is likely that chronic elevation of oxidative stress will modify and alter the structure of myelin proteins and have negative impacts on myelin structure and nerve function in peripheral neuropathies. Among the various oxidative modifications of proteins, carbonylation is a critical and irreparable modification that has been shown to be elevated during aging, diabetes and in neurodegeneration [12], [13], [14], [15], [16], [17], [18]. Diabetic subjects are known to have elevated glucose-, and lipoxidation-derived carbonyl stress [18], [19], [20], [21], [22], [23], however, it remains unknown if carbonylation of sciatic nerve/myelin proteins are elevated in peripheral neuropathies. Therefore, we asked (i) does oxidative stress have an effect on carbonylation and misfolding of sciatic nerve proteins in peripheral neuropathy, and (ii) is the carbonylation of sciatic nerve/myelin proteins associated with loss in nerve conduction and myelin in peripheral neuropathy? We chose two distinct models to address the questions, the well-characterized leptin receptor deficient (*dbdb*) mouse model of diabetic polyneuropathy [2], [24], and mice lacking the superoxide dismutase 1 (Sod1) gene (*Sod1^−/−^*) as a model of *in vivo* oxidative stress [25], [26], [27], [28]. Sod1 is an important cytosolic antioxidant enzyme that detoxifies harmful superoxide radicals. Previous studies from our group have shown that *Sod1^−/−^* mice exhibit significant increase in *in vivo* oxidative stress and deficits in neuromuscular function with age [25], [26], [27], [28]. However, it remains unknown if the chronic elevation of oxidative stress in *Sod1^−/−^* mice results in alterations in myelin structure and reduced nerve conduction and whether these phenotypes are linked to increase in sciatic nerve protein carbonylation and misfolding.

It is well established that oxidative modification of proteins and their subsequent misfolding can lead to their aggregation, which may have an important role in the pathology of several diseases including neurodegenerative diseases [29], [30], [31]. With respect to the peripheral nervous system, it has been shown that peripheral myelinating protein 22 (PMP22), a key myelin protein, undergoes mutation and aggregation in multiple mouse models of human neuropathies [32], [33], [34], [35]. In fact, aggregation of PMP22 has been proposed to be causal for the demyelination phenotype in the *J trembler* mouse model of human peripheral neuropathy [32], [35]. However, it remains unknown if the decrements in nerve conduction and myelination in diabetic peripheral neuropathy is linked to carbonylation and aggregation of PMP22.

In this study, we report that reduced nerve conduction and altered myelin morphology in *dbdb* and *Sod1^−/−^* mice are associated with increases in carbonylation and misfolding of sciatic nerve proteins. We further show that PMP22 undergoes carbonylation and aggregation in sciatic nerves of *dbdb* mice, a finding that is supported by our *in vitro* oxidation assay with tert-butyl hydroperoxide (tBHP) and purified PMP22. Our results reveal for the first time that sciatic nerve/myelin proteins are potential targets for carbonylation and aggregation, which may have important implications for loss in myelin integrity and nerve conduction in peripheral neuropathies.

## Results

### Alterations in nerve conduction and myelin structure in *dbdb* and *Sod1^−/−^* mice

**Figure 1:**
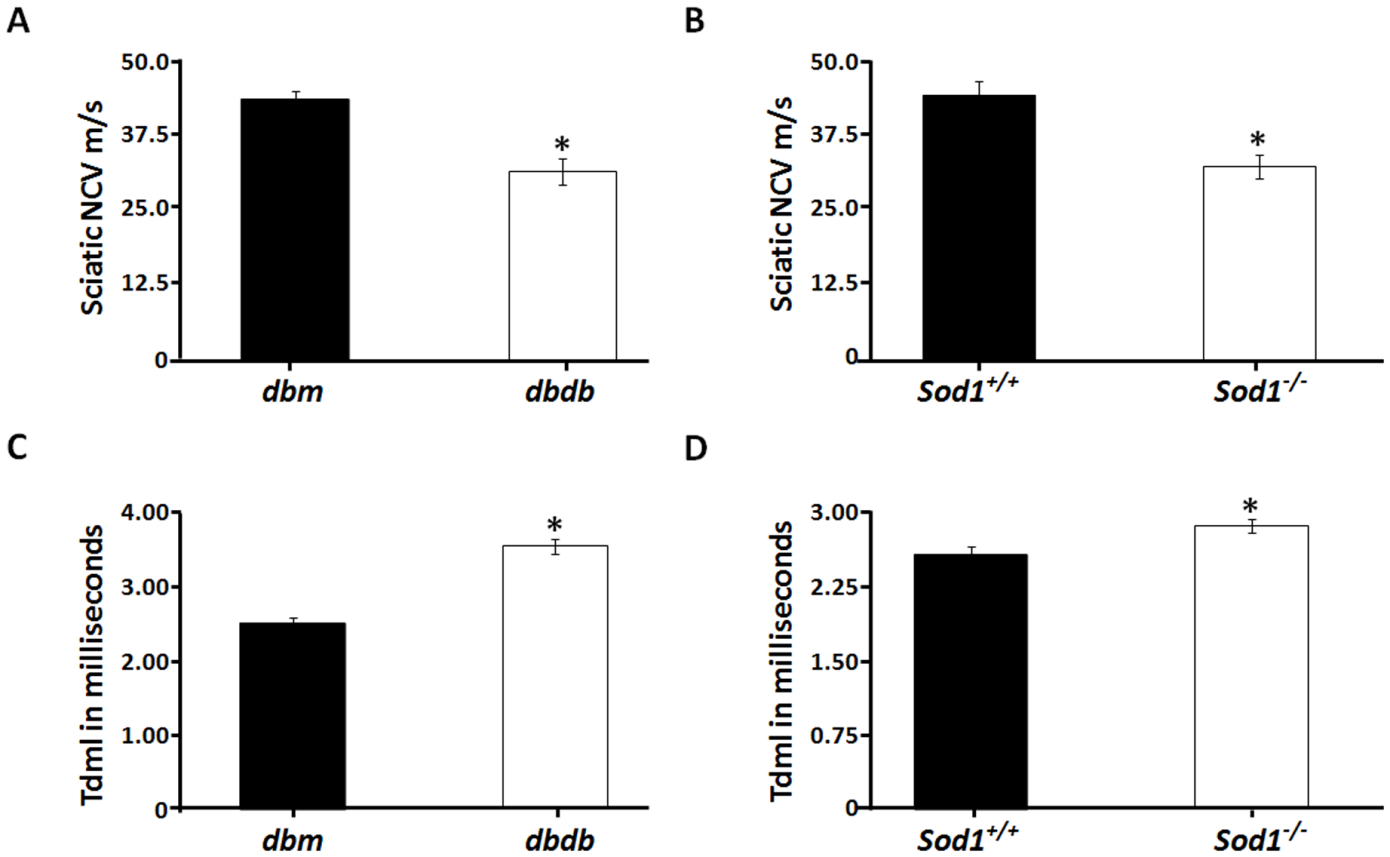
Reduction in peripheral nerve function in *dbdb* and *Sod1* *^−/−^* mice. Electrophysiologic measurements were performed on *dbdb* and *Sod1^−/−^* mice with their corresponding littermate controls. All measurements of nerve conduction velocity (NCV) and latency were performed under isofluorane anesthesia with skin temperatures between 33°C and 34°C. Sciatic NCV was measured in (**A**) 5 month old *dbdb* and (**B**) 6 month old *Sod1^−/−^* mice. Tail distal motor latency was measured in (**C**) *dbdb* and (**D**) *Sod1^−/−^* mice. Statistically significant differences obtained from the different groups (n = 15) were tabulated as follows for the mean ± SEM (*p<0.05 by two-tailed *t-test*).

*Dbdb* mice have been previously shown to have reduced sciatic NCV and increased tail distal motor latency (tdml) [2]. In the current study, we wanted to determine whether oxidative stress is associated with reduced sciatic NCV and increased tdml. We observed a 1.38±0.04 (*p*<0.001) fold reduction in sciatic NCV by 4 months of age in *dbdb* mice (Figure 1A). 6-mo-old *Sod1^−/−^* mice also demonstrated a 1.36±0.07 (*p*<0.01) fold reduction in sciatic NCV (Figure 1B). The *dbdb* mice and *Sod1^−/−^* mice exhibited a 1.41±0.04 fold (*p*<0.001) and 1.12±0.02 fold (*p*<0.05) increase in tdml, respectively (Figure 1C&D).

**Figure 2:**
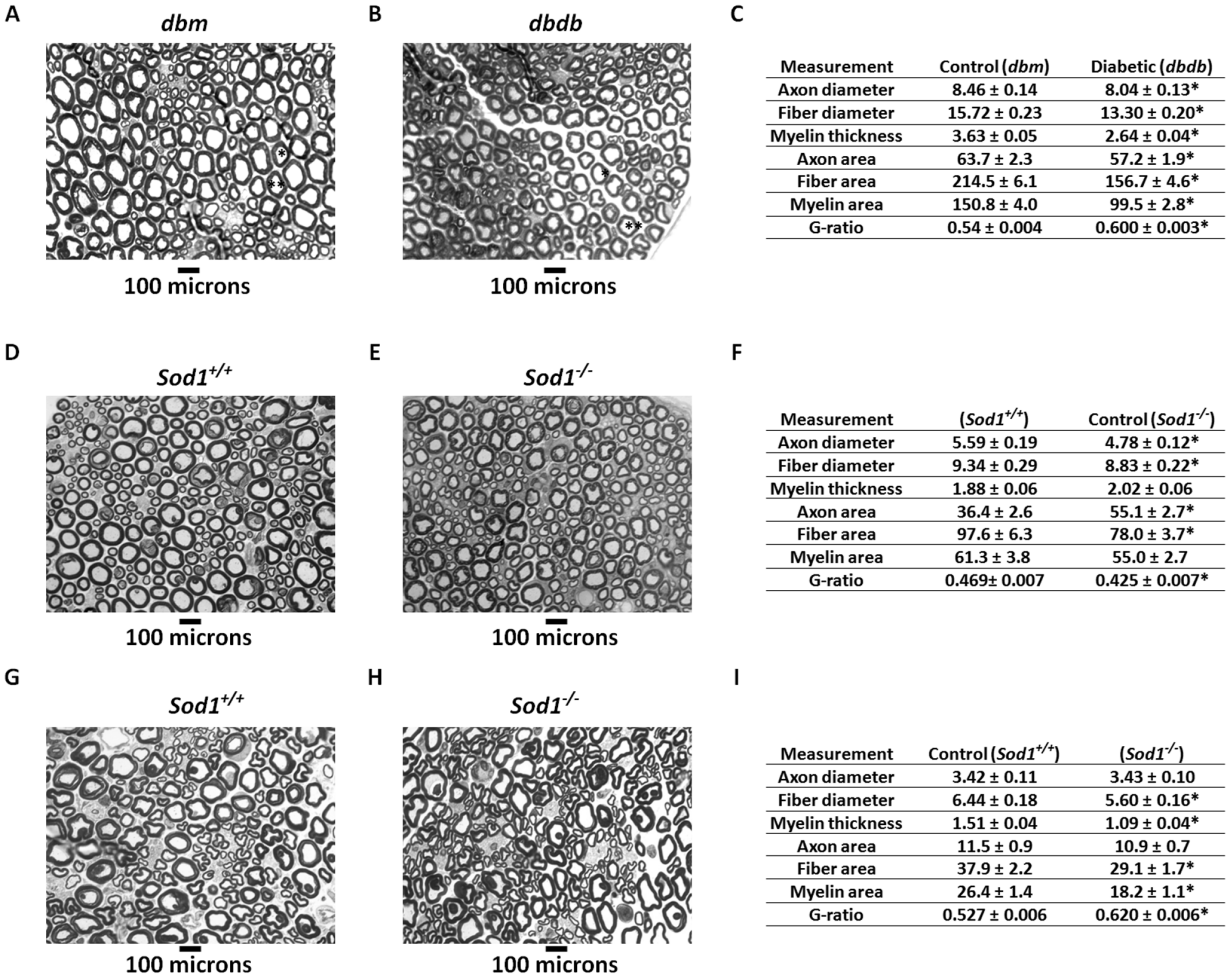
Reductions in myelin and neuron structure in *dbdb* and *Sod1^−/−^* mice. Thick sections of sciatic nerves were sectioned and visualized at 100x (**A**) non-diabetic *dbm* mouse and (**B**) *dbdb* mice. (**C**) Quantification of axon diameter/area, nerve fiber diameter/area, myelin area/thickness and g ratio (axon diameter/fiber diameter) were tabulated from thick sections of *dbdb* and control mice (n  = 3). Thick sections of (**D**) 6-mo-old control and (**E**) 6-mo-old *Sod1^−/−^* mice are shown. (**F**) Quantification was performed similarly to that shown in 2C. Thick sections of (**G**) 20-mo-old control and (**H**) 20-mo-old *Sod1^−/−^* mice are presented. (**I**) Quantification was performed similarly to that in 2C and results expressed as mean ± SEM of n = 3 mice/group. Results were analyzed by two-tailed *t-test* (*p<0.05 by two-tailed *t-test*).

Since demyelination is closely linked to reduced nerve conduction [2] and oxidative stress is associated with reduced sciatic NCV and increased tdml as shown in Figure 1A–D, we measured axon diameter, nerve fiber diameter and myelin thickness in 5-mo-old *dbm* and *dbdb* mice, and 6 and 20 mo-old *Sod1^−/−^* mice in toluidine blue stained sciatic nerve thick sections. *Dbdb* mice exhibited reduced axon, nerve fiber diameter and reduced myelin thickness when compared to comparable sized axons from sections obtained from the sciatic notch in *dbm* mice (Figure 2 A&B, marked by asterisks). Moreover, a significant reduction in axon diameter/area (*p*<0.05), fiber diameter/area (*p*<0.001), myelin thickness/area (*p*<0.001) and increase in G-ratio (axon diameter/fiber diameter) (*p*<0.001, Figure 2C) was also observed in these sections. Reductions in axon diameter, fiber diameter and myelin thickness in *dbdb* mice are consistent with previous studies [2]. In comparison, the *Sod1^−/−^* mice exhibited reduced axon and fiber diameters compared to age-matched wild-type mice at 6 months of age (Figure 2D&E), which was confirmed by quantification of axon diameter/area (*p*<0.001) and fiber diameter/area (*p*<0.001). The myelin area was reduced but the results did not reach statistical significance (*p* = 0.16) (Figure 2F). However, 20 month *Sod1^−/−^* mice showed reductions in myelin thickness/area and fiber diameter/area (Figure 2G&H). Quantification of nerve morphology demonstrated a significant reduction in fiber diameter/area (*p*<0.001), and myelin thickness/area (*p*<0.001) with concomitant increase in G-ratio (*p*<0.001), by 20 months of age (Figure 2). These results strongly suggest that oxidative stress is closely associated with alterations in peripheral nerve myelin morphology and function.

### Protein carbonylation and misfolding in *dbdb* and *Sod1^−/−^* mice

**Figure 3:**
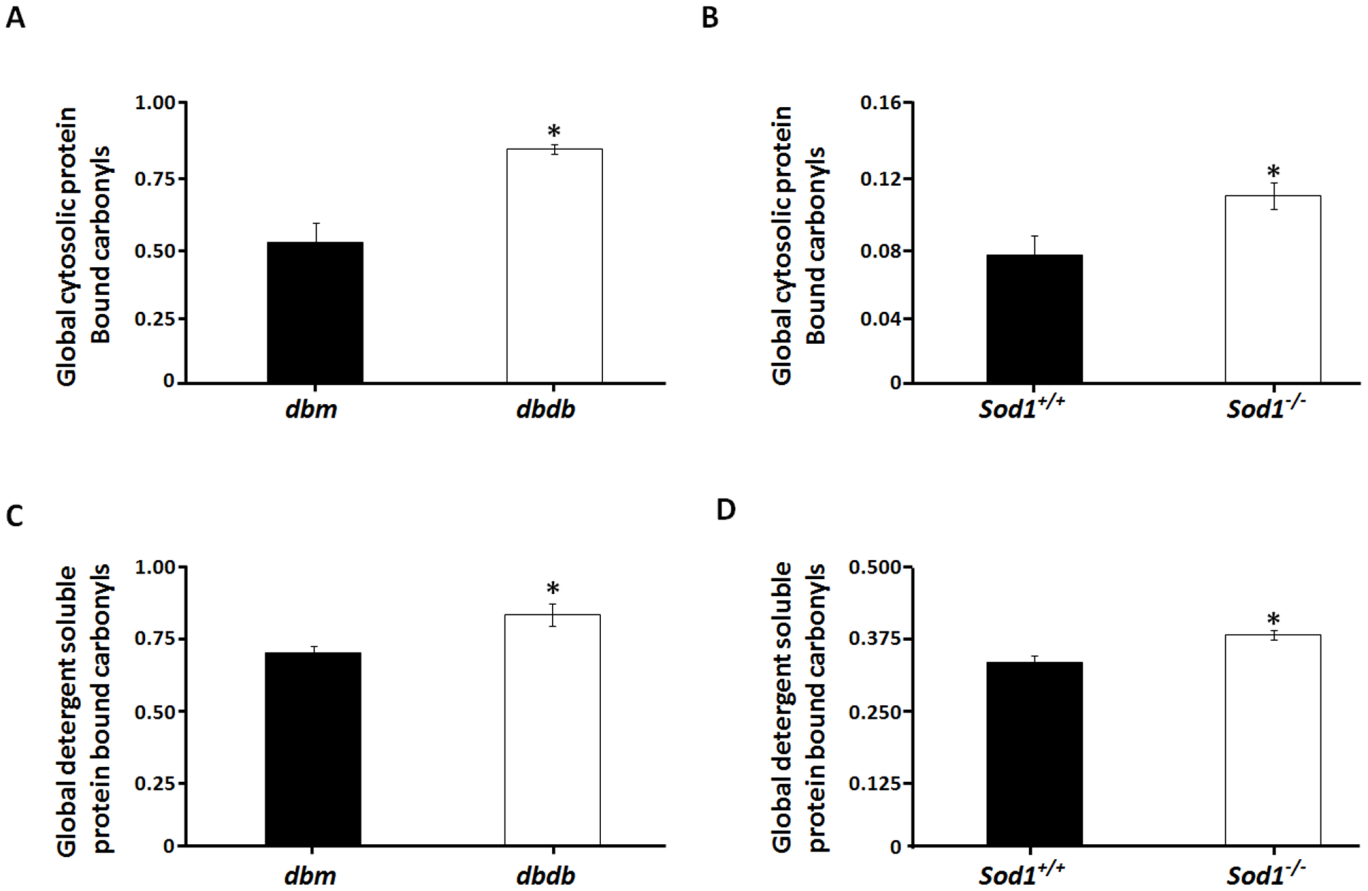
Increase in sciatic nerve protein carbonyls in *dbdb* and *Sod1^−/−^* mice. Total sciatic nerve cytosolic protein bound carbonyls in (**A**) *dbdb* and (**B**) *Sod1^−/−^* mice are presented. Total protein bound detergent-soluble protein carbonyls in (**C**) *dbdb* and (**D**) *Sod1^−/−^* mice are shown. Results are expressed as mean ± SEM (n = 6; *p<0.05 by two-tailed *t-test*).

Our findings on impaired nerve conduction and reduced myelin thickness in *dbdb* and *Sod1^−/−^* mice strongly suggest that oxidative stress might be the common contributor for these changes. In fact, other studies have indicated that oxidative stress might be the causative factor for nerve fiber abnormalities in diabetes [4], [5]. We have previously shown that oxidative stress has major impact to the structure and function of proteins and enzymes [36]. Therefore, we investigated if protein oxidation and misfolding are selectively elevated in both the experimental models. Protein carbonylation is one of the common oxidative modifications detected and studied in aging [14], [37], [38], [39] and in various patho-physiological conditions including diabetes [40]. In this study, we asked if protein carbonylation has any negative impact to structure of proteins in sciatic nerves of *dbdb* and *Sod1^−/−^* mice. We measured protein carbonylation and exposure of hydrophobic domain of sciatic nerve proteins utilizing two distinct fluorescence-based technologies developed by our group [14], [41]. We found a significant increase in the overall level of protein carbonyls in the cytosolic fraction of both *dbdb* mice (1.7±0.2 fold, *p*<0.01, Figure 3A) and *Sod1^−/−^* mice (1.3±0.1 fold, *p*<0.05, Figure 3B). This intriguing observation led us to examine if the protein carbonylation level is also elevated in the detergent-soluble fraction as it has been often found that protein oxidation, including protein carbonylation induces protein aggregation [42], [43], [44]. Therefore, we measured protein carbonylation in the detergent-soluble protein fraction isolated from the sciatic nerve of *dbdb* and *Sod1^−/−^* mice. As we predicted, protein carbonyls were elevated 1.30±0.10 fold in *dbdb* mice (*p*<0.05, Figure 3C) and 1.24±0.08 fold (*p*<0.05, Figure 3D) in *Sod1^−/−^* mice compared to their respective controls.

**Figure 4:**
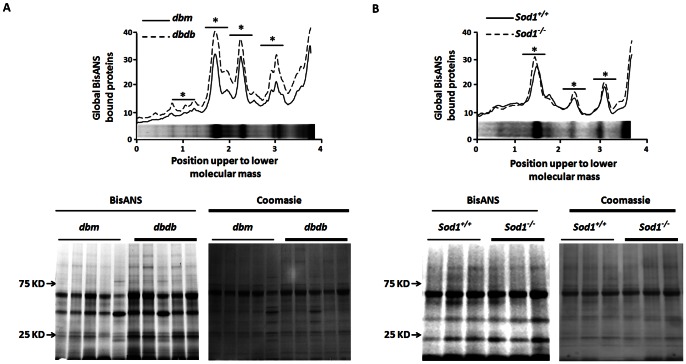
Increase in hydrophobic domain of protein exposure in *dbdb* and *Sod1^−/−^* mice. Total BisANS labeling of sciatic nerve homogenates for (**A**) *dbdb* and (**B**) *Sod1^−/−^* mice are presented. Results are expressed as mean ± SEM (n = 6; *p<0.05 by two-tailed *t-test*).

Since both cytosolic and detergent-soluble fractions exhibited elevation in protein carbonylation, we next asked whether the global state of protein conformation is affected in these experimental models by measuring protein hydrophobicity using a BisANS photolabeling approach. There was a 1.8±0.1 (*p*<0.001) fold increase in global exposure of hydrophobic pockets in sciatic nerves of *dbdb* mice (Figure 4A) and a 1.26±0.03 fold increase (*p*<0.05, Figure 4B) in *Sod1^−/−^* mice.

### PMP22 is carbonylated and aggregated in *dbdb* sciatic nerves

**Figure 5:**
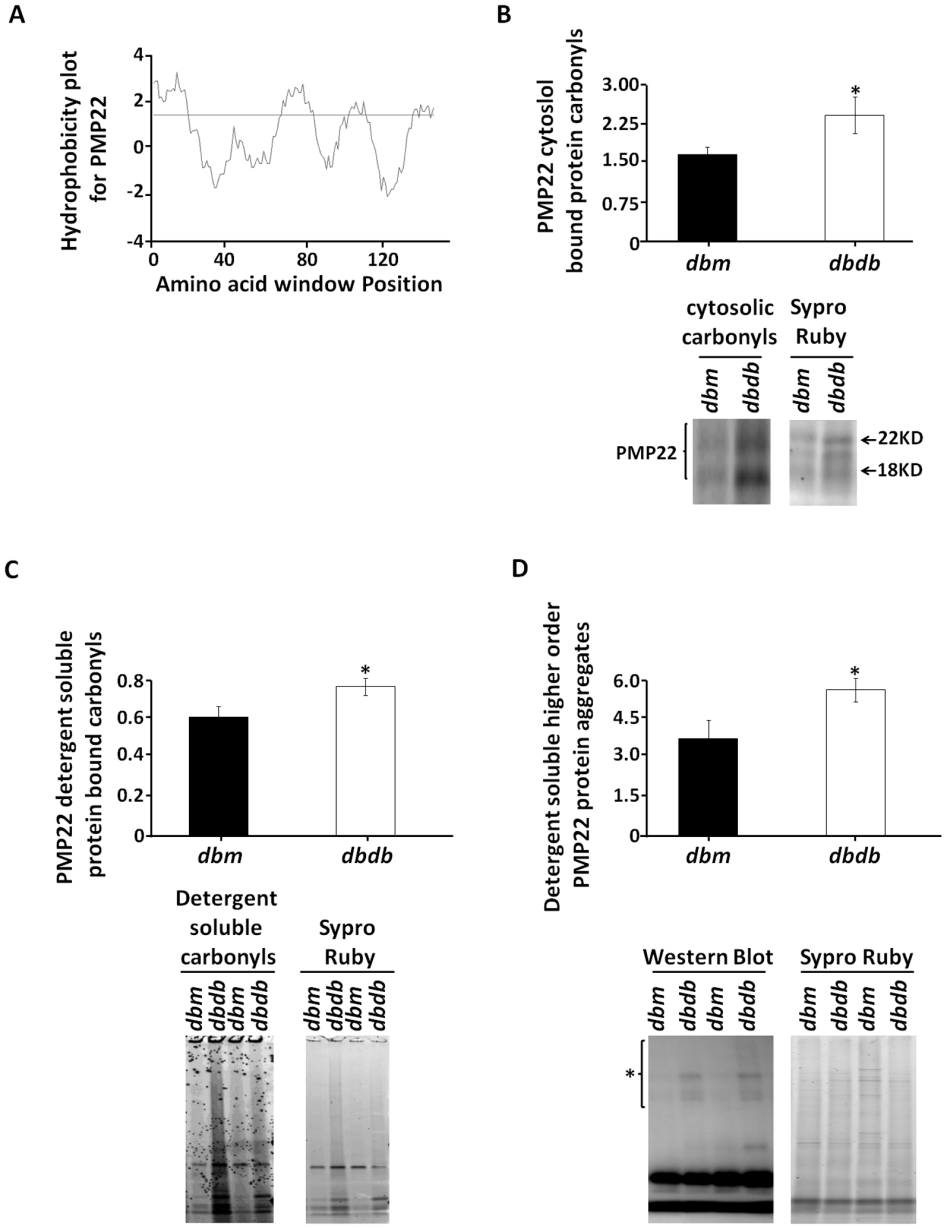
Determination of PMP22 protein carbonylation, changes in hydrophobicity, and aggregation in *Dbdb* mice. (**A**) A Kyte-Doolittle Hydropathy plot was determined for PMP22 hydrophobicity. FTC labeled (**B**) cytosolic and (**C**) detergent-soluble protein fractions were immunoprecipitated with anti-PMP22 polyclonal antibody, loaded onto SDS-PAGE gels and analyzed for protein carbonylation. (**D**) The detergent soluble fraction was analyzed by Western blot against PMP22. The higher order PMP22 aggregates are indicated by *. Results are expressed as mean ± SEM (n = 6; *p<0.05 by two-tailed *t-test*).

Because we found a global increase in cytosolic and detergent-soluble sciatic nerve protein carbonyls in *dbdb* mice and increase in global exposure of hydrophobic pockets, we next investigated if peripheral myelin proteins undergo carbonylation and aggregation in diabetic neuropathy. We chose PMP22 to test our hypothesis as it is one of the abundant proteins (2–5%) in peripheral myelin [11]. Moreover, PMP22 has been found as a key player in multiple Charcot Marie Tooth Disease 1a neuropathies and has been reported to be mutated and aggregated in several neuropathies [32], [33], [34], [35]. We first asked whether the primary sequence of PMP22 can predict and identify any domain that have motif(s) of hydrophobicity as hydrophobic domains in general initiate the aggregation process. We utilized the primary sequence of PMP22 obtained from protein search and online software Kyte-Doolittle Hydropathy plots for this theoretical study. We found that certain regions in PMP22 have motifs of hydrophobicity which can predict its region-specific hydrophobicity (Figure 5A). We next determined if PMP22 is preferentially carbonylated in *dbdb* mice and found that immunoprecipitated cytosolic PMP22 is heavily carbonylated (1.7±0.3 fold increase over *dbm*, *p*<0.05, Figure 5B).

Since oxidative modifications often induce protein aggregation, we investigated if oxidized PMP22 can be detected in the detergent-soluble fraction selectively in *Dbdb* mice and also to determine if PMP22 undergoes higher-order aggregation state. We found that the detergent-soluble PMP22 is heavily carbonylated in *dbdb* mice (1.31±0.12 fold increase over *dbm*, *p*<0.05, Figure 5C). Also, we observed a higher-order aggregation state of PMP22 (1.6±0.2 fold increasincrease) in *Dbdb* mice (p<0.05, Figure 5D).

**Figure 6:**
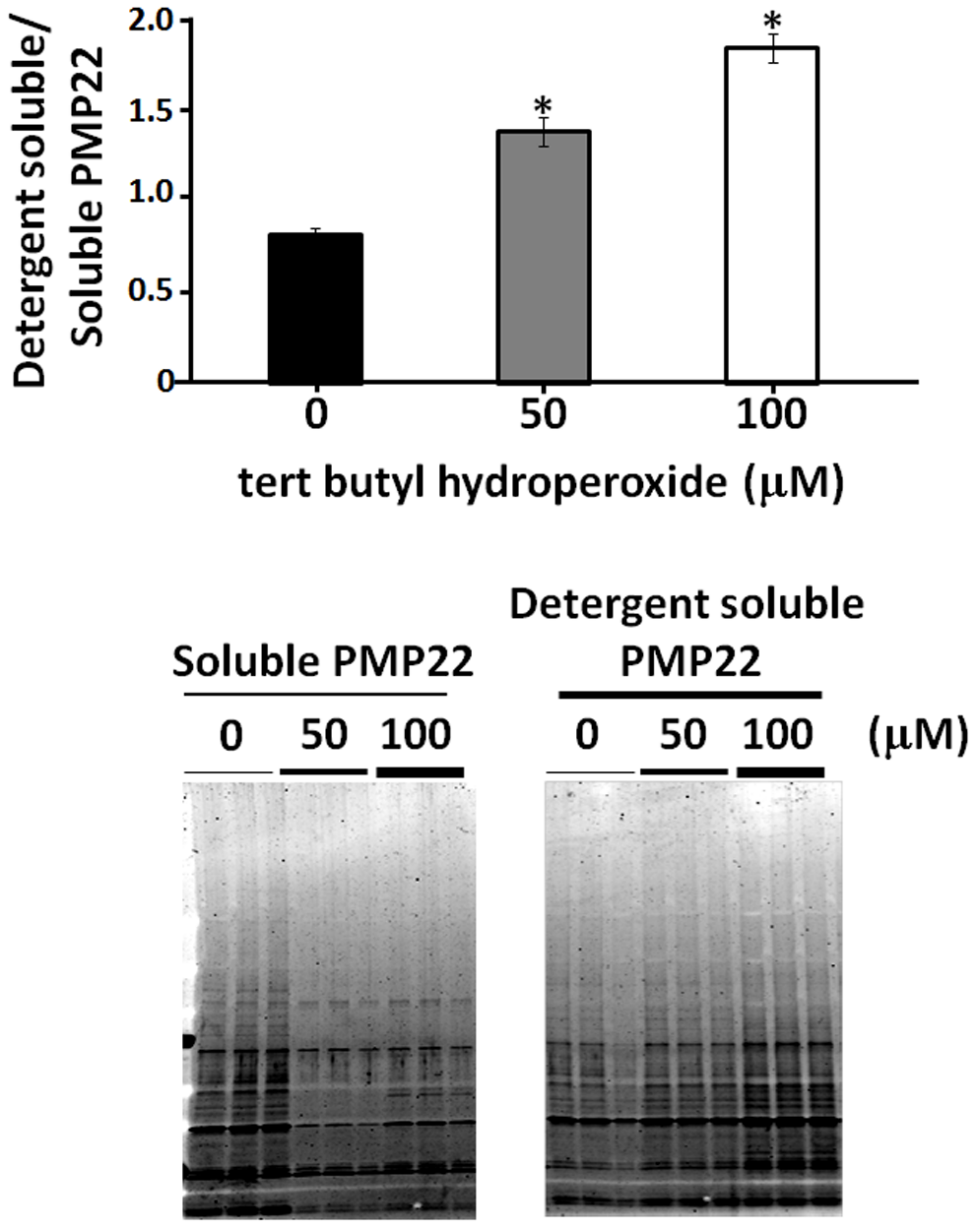
*In vitro* assessment of oxidative stress-induced aggregation of PMP22 protein. Purified PMP22 protein was subjected to *in vitro* oxidation with increasing concentrations of tert-butyl hydroperoxide. SDS-PAGE was performed on oxidized PMP22 protein from both the soluble and detergent-soluble protein fractions. Results are expressed as mean ± SEM (n = 6; *p<0.05 by two-tailed *t-test*).

Based on these intriguing *in vivo* observations, we predicted that the oxidative environment is likely to induce aggregation of PMP22 protein which has been found as aggregates in multiple PMP22 aggregation-dependent neuropathies. Therefore, we exposed purified PMP22 to different concentrations of tBHP (which generates hydroxyl radical and forms protein carbonyls) and then followed the structural consequences of PMP22. The results of Figure 6 shows a dose-dependent increase in insoluble to soluble ratio of PMP22, which confirms our *in vivo* finding that oxidative stress indeed increases the sensitivity of PMP22 to undergo aggregation.

## Discussion

In humans, the diabetic etiology includes neuronal dysfunction, axonal degeneration, Schwann cell dysfunction, and chronic motor/sensory neuron demyelination with increasing disease severity [1]. It has been reported that Schwann cell death [45], neuronal cell death as observed in streptozotocin-induced diabetes [46], and damage to motor/sensory neurons [46] are associated with diabetic neuropathy and reduced nerve function. All of these changes are likely contributors to loss of muscle mass, limb pain and dysfunction of the lower extremities in *dbdb* mouse model of peripheral neuropathy. Chronic oxidative stress is believed to be one of the leading underlying mechanisms of reduced myelination in diabetic neuropathy. Indeed, recent studies have shown that reduction in oxidative stress may improve sciatic nerve/myelin morphology and function in diabetic neuropathy [4], [5]. It has also been well documented that oxidative stress plays a key role in initiating misfolding and aggregation of proteins and enzymes [36], [41], [42], [43], [44], [47]. However, it is unknown whether imbalanced structural states of proteins (protein oxidation, misfolding and aggregation) are linked to reduced nerve conduction and myelin thickness in peripheral neuropathies, in part, due to the lack of sensitive technologies to measure these changes. In this study, we report for the first time that sciatic nerve proteins of *dbdb* and *Sod1^−/−^* mice undergo oxidation (protein carbonylation) and alteration in protein conformation which may be closely linked to decrement in nerve conduction and altered myelin morphology.

The novelty of this study is the use of two mouse models to determine the role of oxidative stress in peripheral neuropathy (i) the well-characterized *dbdb* mouse model of diabetic peripheral neuropathy that is known to be associated with oxidative stress/damage [2], [24] and (ii) the *Sod1^−/−^* mouse model of *in vivo* oxidative stress which exhibits age-associated deficits in neuromuscular function [25], [26], [27], [28]. We found significant changes in protein carbonylation and misfolding in diabetic mice which correlated well with reductions in sciatic NCV and myelin thickness and was in agreement with previous studies in *dbdb* mice [2] and human type II diabetes [3]. *Sod1^−/−^* mice exhibited all of the physiological changes observed in *dbdb* mice (with the exception of myelin thickness) at 6 months of age and biochemical changes (elevation of protein carbonylation and alteration in protein surface hydrophobicity), however, the changes at this age were not as robust as that observed in 5-mo-old *dbdb* mice. It is interesting to note that *Sod1^−/−^* mice also exhibited a reduction in sciatic NCV and a modest increase in tdml, which was also less robust than that observed in *dbdb* mice. There was clearly an age-related effect in *Sod1^−/−^* mice as myelin thickness was significantly reduced by 20 months of age which affirms that oxidative stress is indeed one of the critical determinants for demyelination in peripheral neuropathies. We postulate that the subtle effects on myelin thickness observed in *Sod1^−/−^* mice compared to *dbdb* mice at 6 months of age may be due to a lesser degree of oxidative damage and misfolding in these mice. As both sugar aldehydes (arabinose, glyoxal, methyl glyoxal, glycoaldehyde, and 3-deoxyglucosone) and lipid aldehydes (4-hydroxynonenal, malondialdehyde, glyoxal, and acrolein) form carbonyl adducts to proteins by Michael's addition and are found to be elevated in diabetes [18]–[23], we predict that both sugar and lipid aldehydes are likely the major contributors for elevated protein carbonylation in *Dbdb* mice. Nevertheless, all of these data strongly suggest that oxidative stress might play a critical role in reduction of NCV and alteration in myelin structural integrity.

Since PMP22 is one of the most abundant peripheral myelin proteins (2–5%) [11] and aggregation of PMP22 has been implicated in multiple CMT1a demyelinating neuropathies [34], [35], we asked whether PMP22 is sensitive to carbonylation and aggregation in *dbdb* mice. Diabetic mice exhibited elevated PMP22 carbonylation in both cytosolic and detergent-soluble fractions, as well as formed higher-order aggregates in the detergent-soluble fractions. These findings are important considering that the diabetic nerve morphology after necropsy has similar opaqueness to that of sciatic nerves isolated from *J trembler* mice, a model of CMT1a (PMP22 mutant mice, a model of Hereditary Neuropathy with Liability to Pressure Palsy). In this context, it is also interesting to note that an increase in PMP22 insolubility in *J trembler* mice is causal for the demyelination phenotype [32], [33], [34], [35]. Moreover, a chromosome 22 duplication in humans containing the PMP22 protein increases PMP22 insolubility in mice and reduces PMP22 incorporation in myelin while heat shock improves myelin incorporation [34], [35], [48]. The hydropathy plot analysis performed in this study revealed that the primary structure of PMP22 has four distinct regions in the primary sequence that are more hydrophobic than the rest of the sequence which could explain the propensity of PMP22 to aggregate in *dbdb* mice. It is also important to note that the mutation of leucine19 on PMP22 to proline (first trans-membrane domain) favors β-sheet conformation while the wild-type PMP22 adopts a stable α-helical conformation. This transition from α-helix to β-sheet is likely to favor the aggregation of PMP22 [49]. We predict that carbonylation at this region on PMP22 may shift its conformation from α-helix to β-sheet and promote its aggregation. This can plausibly explain the increase in PMP22 aggregation as a consequence of elevated oxidative stress in both our *in vivo* and *in vitro* studies.

In conclusion, our data indicates that reduction in NCV associated with altered myelin morphology in *dbdb* and *Sod1^−/−^* mice may be a consequence of elevated global protein carbonylation and exposure of surface hydrophobic domain on proteins. Our future studies will determine if the protection of sciatic nerve/myelin proteins from oxidative damage will attenuate the loss in NCV and myelin thickness observed in peripheral neuropathies including diabetic polyneuropathy.

## Materials and Methods

### Materials

Phosphatase and protease cocktail was purchased from Thermoscientific, Rockford, IL. PMP22 polyclonal antibody was purchased from Abcam (ab61220, Abcam, Cambridge, MA). Purified PMP22 was purchased from Novus Biologicals (H00005376, Littleton, CO). BisANS (4,4'-dianilino-1,1'-binaphthyl-5,5'-disulfonic acid, dipotassium salt) and FTC (Fluorescein-5-thiosemicarbaizide) were purchased from Invitrogen (Grand Island, NY).

### Ethics Statement

This study was carried out in strict accordance with the Guide for the Care and Use of Laboratory Animals of the National Institutes of Health's recommendations. All procedures were approved and performed in accordance with the Committee on the Ethics of Animal Experiments (Institutional Animal Care and Use Committee at the University of Texas Health Science Center at San Antonio (UTHSCSA)) under protocol 10003-34-01-A and 08080z as well as the Audie L. Murphy Veterans Hospital in San Antonio using protocol 0503-002. All experiments were performed to minimize pain and discomfort.

### Animals

**a)** Diabetic mice homozygous for leptin receptor mutant(*Lepr^db^* ^+/+^ *Dock7m-J (dbdb)*) and their heterozygous controls (*Lepr^db^* ^+/−^ *Dock7m-J (dbm)*) in a C57BL/KS-J background were purchased from Jackson Laboratories (Bar Harbor, Maine, USA) (Protocol, 10003-34-01-A). All mice were fed an *ad libitum* normal chow diet. Experiments were performed in 5-month-old *dbm* and *dbdb* mice that exhibit significant deficits in sciatic nerve conduction velocity, increases in latencies and alterations in myelin morphology [2]. **b)** *Sod1^−/−^* mice (on a C57BL/6 background) were used as a model of *in vivo* oxidative stress as described previously (protocol, 08080z and 0503-002) [25], [50]. 6-month-old *Sod1^−/−^* and their wild-type (WT) littermates were used for the biochemical studies. 6-month- and 20-month- *Sod1^−/−^* and their WT littermates were used for morphological assessments.

### Nerve Conduction Velocity and latency

Mice were anesthetized with isofluorane and maintained at 34°C with a heating lamp. All experiments were performed with a Nicolet Viking Quest portable EMG apparatus (CareFusion, San Diego, CA, USA) as described previously [51]. Subdermal needle electrodes were cleaned with 70% alcohol between animals. Supramaximal stimulation was delivered with 0.02 millisecond electrical impulses for all experiments. Electrodes were inserted 3 cm apart and the latency of the tail distal motor action potential was measured by proximal to distal stimulation. Sciatic NCV was measured by stimulating proximal ankle electrodes with current and the latency for response at the dorsal digits divided by the distance traveled was measured. Then the stimulating electrodes were placed at the sciatic notch and the latency to the ankle was measured, subtracted from the initial foot ankle latency and divided by the notch to the ankle to obtain values for sciatic NCV.

### Thick sections and imaging

A 1-2 cm segment of sciatic nerve at the sciatic notch for all sectioning was fixed in 4% paraformaldehyde (PFA) and was switched to buffer containing PBS with 4% PFA and 1% glutaraldehyde in 0.1 M sodium cacodylate buffer, post-fixed in 1% osmium tetraoxide and finally in 1% uranyl acetate. Sections were cut at 1–2 mM in thickness and then stained with the following solution of toluidine blue (1 g of toluidine blue, 1 g of borax and 100 mL of water). Using a 0.2 µl filtered syringe filled with prepared toluidine dye 1 drop was applied to thick sections. Slides were placed at 180 degrees on a hot plate for 10 seconds. Samples were washed with water and allowed to dry and then covered with cover slips. Sectioning of sciatic nerves was performed by the UTHSCSA electron microscopy core (San Antonio, TX) and visualized using Nikon Eclipse TE2000-U fluorescence microscope (Nikon Inc.) at 40- and 100X magnification. Axon and fiber diameters/areas were quantified utilizing Roper Scientific software and analyzed as described earlier [2], [52]. The approximate circumference was quantified to determine the area and diameter of both axons and axon plus myelin (fiber). Myelin diameter and area was obtained from the subtraction of axon diameter and area from fiber diameter and area (Fiber-axon = myelin). The myelin thickness was determined by dividing the myelin diameter by 2. G-ratios were determined as the axon/fiber diameter [2], [52]. Greater than 200 nerve fibers were measured for each individual animal and as described earlier [2], [52].

### Measurement of protein carbonyls

Sciatic nerve protein extracts were made by sonication in 20 mM potassium phosphate buffer, pH 6.0 with 0.5 mM MgCl_2_, and 1 mM EDTA as previously described [14]. Homogenates were centrifuged at 100,000x g for 1 hour to obtain the cytosolic fraction. Pellets obtained after centrifugation were resuspended by sonication in P3 buffer (2% SDS, 0.5% NP40, 0.5% deoxycholate at pH 6.0) and centrifuged at 100,000x g for 20 minutes to obtain the detergent soluble fraction. Both the fractions were labeled with FTC to measure global level of protein carbonyls in cytosol and detergent soluble fractions as previously described [14]. Samples were loaded onto 4–15% gels and visualized utilizing the Typhoon 9400 (Amersham, Piscataway, NJ, USA) with excitation at 532 and emission with a 526 SP emission filter. Total carbonylated proteins were analyzed against the abundance of the protein with Sypro Ruby staining [14] and quantified using Un-Scan-it software (Silk Scientific, Orem, Utah, USA).

### Measurement of protein surface hydrophobicity

Sciatic nerves were homogenized in 50 mM tris buffer, pH 7.4, followed by photo-labeling the protein surface hydrophobic domain with BisANS (0.1 mM) under UV light-exposure as previously described [36], [41]. Thereafter, equal amounts of BisANS-labeled proteins were loaded onto SDS-PAGE and visualized on an Alpha Innotech FluorChem HD2 camera utilizing UV transillumination. The level of incorporation of BisANS was measured as described in protein carbonyls and normalized to Coomassie protein stain [36], [41].

### Determination of hydrophobicity based on primary sequence

Primary sequence for PMP22 was obtained from known sequences on Pubmed protein search for mouse and analyzed for hydrophobicity utilizing Kyte-Doolittle hydropathy plots as described previously [53].

### Immunoprecipitation

FTC-labeled cytosolic and detergent soluble protein fractions were incubated with PMP22 polyclonal antibody (Abcam, Cambridge, MA, Prod# ab61220) in KEI buffer overnight at 4°C. After overnight incubation, 25 µL of protein A bead (Pierce, Prod# 20366) was added and incubated with rotation for 2 hours at 4°C. Samples were then centrifuged at 16000x g for 1 minute. Pellet was washed three times with 500 µL of KEI buffer plus 0.5 M NaCl and then two times with 500 µL of 50 mM Tris. The pellet was dried and 4x loading buffer and 4 mM dithiothreitol were added to the beads. Total carbonyls and protein were measured by SDS-PAGE and quantified as described in the carbonyl assay section.

### Measurement of PMP22 aggregates

Sciatic nerves were homogenized in phosphate buffer, pH 6.0, as described in protein carbonyl measurement section and centrifuged at 100,000xg for 1 hour. Resultant pellets were resuspended by sonication in P3 buffer (2% SDS, 0.5% NP40, 0.5% deoxycholate, pH 6.0) and centrifuged for 20 minutes at 100,000x g to obtain the detergent soluble fraction. One tenth of a µg of protein was used to quantify the total increase in PMP22 in the detergent soluble fraction by western blot using the PMP22 polyclonal antibody. Blots were visualized and scanned on a Typhoon 9400 followed by TMB colorimetric assay (Vector Laboratories, Burlingham, CA) and a Alpha Innotech FluorChem HD2 camera was used to capture the image. Western blot image for the high molecular weight aggregates (75 Kd–250 kd) were quantified.

### *In vitro* oxidation of PMP22

Purified PMP22 was incubated with varying concentrations of tBHP (0, 50, and 100µM) at 37°C for 2 hr. The soluble and pellet fractions of PMP22 were obtained with centrifugation at 100,000x g. The pellet was resuspended in P3 buffer to obtain the detergent-soluble fraction. Soluble and detergent-soluble fractions of PMP22 were run on SDS-PAGE followed by Coomassie stain to quantify PMP22 protein loading. The ratio of soluble to detergent-soluble fraction of PMP22 was quantified.

### Statistical analysis

Results are expressed as mean ± SEM. Significant differences were established utilizing student’s *t-test* and by analysis of the variance with tukey posthoc analysis (ANOVA) utilizing Graph Pad Prism software (La Jolla, CA).
